# High security and privacy protection model for STI/HIV risk prediction

**DOI:** 10.1177/20552076241298425

**Published:** 2024-11-21

**Authors:** Zhaohui Tang, Thi Phuoc Van Nguyen, Wencheng Yang, Xiaoyu Xia, Huaming Chen, Amy B. Mullens, Judith A. Dean, Sonya R Osborne, Yan Li

**Affiliations:** 1 School of Mathematics, Physics and Computing, Centre for Health Research, 7932 University of Southern Queensland, Toowoomba Campus, QLD, Australia; 2 Department of Information Technology, Thanh Do University, Hanoi, Vietnam; 3 School of Computing Technologies, 5376 RMIT University, Melbourne, VIC, Australia; 4 School of Electrical and Computer Engineering, The University of Sydney, Darlington, NSW, Australia; 5 School of Psychology and Wellbeing, Centre for Health Research, Institute for Resilient Regions, 7932 University of Southern Queensland, Ipswich, QLD, Australia; 6 School of Public Health, Faculty of Medicine, The University of Queensland, Herston Campus, QLD, Australia; 7 School of Nursing and Midwifery, Centre for Health Research, Institute for Resilient Regions, 7932 University of Southern Queensland, Ipswich, QLD, Australia

**Keywords:** Homomorphic encryption, federated learning, centralized learning, deep learning, public health, sexually transmissible infections, human immunodeficiency viruses

## Abstract

**Introduction:**

Applying and leveraging artificial intelligence within the healthcare domain has emerged as a fundamental pursuit to advance health. Data-driven models rooted in deep learning have become powerful tools for use in healthcare informatics. Nevertheless, healthcare data are highly sensitive and must be safeguarded, particularly information related to sexually transmissible infections (STIs) and human immunodeficiency virus (HIV).

**Methods:**

We employed federated learning (FL) in combination with homomorphic encryption (HE) for STI/HIV prediction to train deep learning models on decentralized data while upholding rigorous privacy. The dataset included 168,459 data entries collected from eight countries between 2013 and 2018. The data for each country was split into two groups, with 70% allocated for training and 30% for testing. Our strategy was based on two-step aggregation to enhance model performance and leverage the area under the curve (AUC) and accuracy metrics and involved a secondary aggregation at the local level before utilizing the global model for each client. We introduced a dropout approach as an effective client-side solution to mitigate computational costs.

**Results:**

Model performance was progressively enhanced from an AUC of 0.78 and an accuracy of 74.4% using the local model to an AUC of 0.94 and an accuracy of 90.7% using the more advanced model.

**Conclusion:**

Our proposed model for STI/HIV risk prediction surpasses those achieved by local models and those constructed from centralized data sources, highlighting the potential of our approach to improve healthcare outcomes while safeguarding sensitive patient information.

## Introduction

Security and privacy are significant considerations in the context of sexually transmissible infections (STIs) and human immunodeficiency virus (HIV) risk prediction powered by artificial intelligence (AI). Security and privacy protection of an AI-driven STI/HIV risk prediction system should safeguard individuals’ sensitive health information, protect against discrimination, build trust, and ensure compliance with legal and ethical standards.^1,2^ A meticulous balance between the benefits of AI and ensuring robust measures for privacy and security is necessary to achieve meaningful progress in AI-based healthcare systems, particularly in the domain of risk prediction for promoting STI/HIV prevention and treatment. While the application of AI in healthcare is burgeoning in recent decades, utilizing data to build models to predict the likelihood of developing some diseases has significantly improved diagnosis and treatment in clinical settings.^3,4^ Machine learning and deep learning models have demonstrated the potential to forecast the occurrence and patterns of certain infectious diseases.^5,6^ By integrating various machine learning techniques, accurate and credible outcomes can be achieved. The centralization of data management heightens the vulnerability to unauthorized access, data breach, or information misuse. Individuals may exhibit reluctance to share their health information if they perceive there is a risk of compromise in a centralized repository, potentially resulting in privacy violations. Moreover, individuals may be less willing to participate in research or share health data if they perceive a potential threat to personal information being traced back to them. Additionally, the sharing of data must adhere to legal and ethical standards, as exemplified by laws such as the Health Insurance Portability and Accountability Act (HIPAA) of 1996 in the United States.^6^ HIPAA is designed to safeguard patient information. Centralized storage poses challenges in ensuring compliance with these regulations, and any breaches in compliance may expose institutions and researchers to legal consequences, thereby leading to a loss of trust among stakeholders and potential legal action.

When delving into the usage of AI for predicting patient outcomes, the priority focus is to ensure privacy protection.^7,8^ Federated learning (FL) has gained significant interest in healthcare as FL enables clients (personal devices, private clinics, or hospitals) to share their trained local model for the construction of a global model on the server side. The global model is then distributed to each client for outcome predictions. FL facilitates collaboration among healthcare organizations in developing deep learning models without the need to share raw patient data, ensuring privacy preservation for each client.^9,10^

FL provides several significant benefits when implemented in healthcare settings, effectively addressing many of the challenges associated with the sharing of sensitive medical data, such as data relating to clients’ STI/HIV status, while enabling advanced applications of deep learning and AI. Beyond privacy preservation, FL reduces data exposure by retaining data on local devices or servers, minimizing the risk of data breaches or unauthorized access and enhancing data security and confidentiality. Since FL is not required to share sensitive patient data directly, it fosters knowledge sharing and collaborative efforts for developing medical solutions.^8,11^ By aggregating knowledge from a wide range of healthcare centers, FL enables the creation of more robust and generalizable prediction models.^12^ In addition, within the FL system, only the models are shared, reducing the need to transfer large amounts of data over networks. This significantly enhances the overall efficiency and privacy of communication within the system.^13^

While FL offers enhanced privacy protection compared to centralized data solutions, it remains susceptible to specific types of attacks, including model inversion and membership inference attacks.^14,15^ Combining FL with homomorphic encryption (HE) has emerged as a promising solution, not only in the context of the Internet of Things (IoT) healthcare systems^16^ but also in various FL systems such as reported in several papers.^17–21^ In a study conducted by Zhang et al.,^16^ FL was implemented using the HAM10000 medical dataset to address the challenge of skin lesion classification. Additionally, they introduced enhanced federated learning homomorphic encryption (FLHE) solutions and applied them to the HAM10000 medical dataset, revealing improved results compared to conventional methods. However, it is worth noting that there remains a considerable gap in research concerning the application of FL in conjunction with encryption techniques for STI/HIV risk prediction. Moreover, FLHE encounters challenges related to the model's performance and computational overhead.^16^ To overcome these obstacles, in this study, we propose a framework that significantly enhances model accuracy and computation overhead. The summary of our contributions is as follows:

1. We propose an innovative approach, employing FL in combination with HE for STI/HIV prediction, offering a robust solution for training deep learning models on decentralized data while upholding rigorous privacy.
2. An innovative strategy based on two-step aggregation is presented to enhance the model performance, by leveraging the area under the curve (AUC) and accuracy metrics. The two-step aggregation involved a secondary aggregation at the local level before utilizing the global model for each client.
3. We introduce a dropout approach as an effective client-side solution to mitigate computational costs. In this method, each client is assigned a model quality threshold, specifically measured by AUC. If a client's model falls below the AUC threshold, it does not need to be encrypted and transmitted to the server for aggregation.
4. We compare our method with various methodologies for constructing deep learning models used in STI/HIV prediction. Furthermore, a comprehensive evaluation of the advantages and disadvantages of each solution was performed, providing a valuable reference point for future applications.

The remainder of the paper is constructed as follows. In Literature review section, related work about FL and encryption is briefly surveyed. In Fundamentals of federated learning and homomorphic encryption in STI/HIV risk prediction section, FL for digital health applications and the principles of HE are discussed. Methods section presents the methods for development of the proposed system, including the scheme and different strategies to enhance model performance and computational cost. Results section reports the results of the performance evaluation of our proposed solution. We conclude the paper in Conclusion section with a discussion of future works.

## Literature review

We first discuss the literature about FL and HE for healthcare applications. In AI for healthcare, issues related to privacy and data protection often result in data isolation. When models are exclusively built on isolated data islands, there is a risk of missing valuable insights and knowledge. These data-sharing limitations can hinder the progress of AI applications, causing a slowdown in their development.^16^ To address privacy concerns and improve model quality, Google introduced the concept of FL, known as Federated Average (FedAvg), in 2016. This approach aimed to optimize the efficiency of machine learning models for smartphones while simultaneously ensuring robust privacy protection for personal devices.^7,22,23^

FL has experienced continuous development through various studies. A recent contribution by Houssein and Sayed^7^ introduced a novel approach called FedImpPSO to overcome the accuracy challenges encountered by current FL methods in unstable networks due to the substantial weight data volume. FedImpPSO improved algorithmic robustness in unpredictable network conditions by aggregating score values from FL models and utilizing an enhanced version of particle swarm optimization (PSO). The study further extended the application of FedImpPSO to the healthcare domain, demonstrating its effectiveness through two case studies. In the first case, COVID-19 classification using ultrasound and X-ray datasets achieved F1 measures of 77.90% and 92.16%, respectively. In the second case, focused on cardiovascular data, FedImpPSO achieved accuracy rates of 91.18% and 92% in predicting the presence of heart diseases.

While FL has demonstrated superior performance, it faces the emergence of diverse adversarial attacks.^24^ A comprehensive exploration of reconstruction attacks and their countermeasures were presented in a study by Bhowmick et al.^25^ Zhu et al.^26^ introduced a depth gradient leakage scheme, enabling adversaries to reconstruct images closely resembling the original sample solely based on the local model when additional information is not available. This highlights the vulnerability in FL. Even if the original data is kept locally, updating the local model can still create opportunities for potential adversarial attacks.

In response to the rise of many attack models that pose threats to the privacy and confidentiality of machine learning, several protective measures have been proposed. Hardy et al.^17^ and Zhang et al.^21^ utilized the additive HE algorithm to shield the local model from being observed by curious participants during the model aggregation process. HE offers a robust assurance of privacy preservation.

The HE is also used in the work by Jing et al.^18^ They introduced xMK-CKKS, an enhanced iteration of the MK-CKKS multi-key HE protocol. This protocol served as the foundation for a novel privacy-preserving FL approach. In their method, model updates were encrypted using a combined public key before being shared with a central server for aggregation. Decryption necessitates collaboration among all participating devices. Their approach effectively protected against privacy breaches arising from the public sharing of model updates in FL, and it exhibits resilience against any collusion attempts between the participating devices and the server. Through their evaluations, they demonstrated that their scheme outperformed other recent advancements in terms of communication and computational efficiency while maintaining the accuracy of the model.

The integration of FL and HE was also explored in the work by Fang et al.^27^ This work combined HE and FL to ensure the security of both data and models throughout the training process. Furthermore, the combination of HE and FL maintains data privacy when multiple parties collaborate. Their proposed algorithm achieved a similar level of accuracy in model training as traditional methods. Through experiments conducted on datasets such as MNIST and mental fatigue, the accuracy difference was maintained below 1%. Concerning the computational cost associated with HE, the study by Feng et al.^27^ assessed the impact of varying key lengths and network complexities. The analysis revealed that increasing the key length or complexity of the network leads to higher computational overhead. Fang et al.^27^ emphasized the significance of finding a pragmatic solution to balance the trade-off between performance and security.

The application of FLHE for the IoT-based healthcare system was presented by Zhang et al. in.^16^ Cryptographic techniques such as masks and HE were implemented to enhance the protection of local models, preventing adversaries from extracting private medical data through threat actions, including model reconstruction or model inversion. The primary determinant of a local model's contribution to the global model during each training epoch was not the size of the dataset, as typically used in deep learning, but rather the quality of the datasets possessed by different participants. Additionally, a dropout-tolerant approach was introduced, ensuring that the FL process continues if the number of online clients remains above a preset threshold. The security analysis confirmed the effectiveness of their proposed scheme in safeguarding data privacy. Moreover, a theoretical investigation of the computational and communication costs was conducted. As a practical example within the healthcare domain, the scheme is applied to the classification of skin lesions using training images from the HAM10000 medical dataset. Experimental results demonstrated that the proposed approach yields promising outcomes while maintaining privacy.

As a summary of the literature review, FL and HE exhibit significant potential for applications in the healthcare sector. Note that there are some existing research works (e.g., Ref.^28^) that used differential privacy for sensitive data protection. However, differential privacy achieves privacy by adding some noise to the data or the output of any query. This added noise decreases the utility and accuracy of data analysis. Suppose the data is sensitive and requires a strict guarantee of privacy. In this case, the added noise becomes large enough to cause degradation in the performance of models and analyses driven by data. For the above reasons, the differential privacy technique is excluded from this work.

## Fundamentals of federated learning and homomorphic encryption in STI/HIV risk prediction

In this section, we provide the fundamentals of FL and HE.

### Federated deep learning for STI/HIV risk prediction

There are some traditional deep learning architectures, such as convolutional neural networks (CNNs), recurrent neural networks (RNNs), and feedforward neural networks (FNNs). These network models comprise various layers, including the input layer, hidden layer, and output layer. The layers are connected via neurons, which are defined by activation functions, weights, and biases. The choice of artificial neural network models depends on the type of data being processed. For example, CNNs are primarily designed for processing grid-like data, such as images and sequences, where the spatial relationships between data points are important.^29^ They are not commonly employed for processing discrete tabular data, as CNNs may not be well suited for capturing tabular data's inherent relationships and patterns.

Similarly, RNNs are tailored for sequential data, such as time series or natural language text, where the order of data points is of paramount importance.^30^ However, in tabular data, where the order of rows or columns generally lacks meaningful information, RNNs may not effectively leverage their sequential processing capabilities.

**Figure 1.**
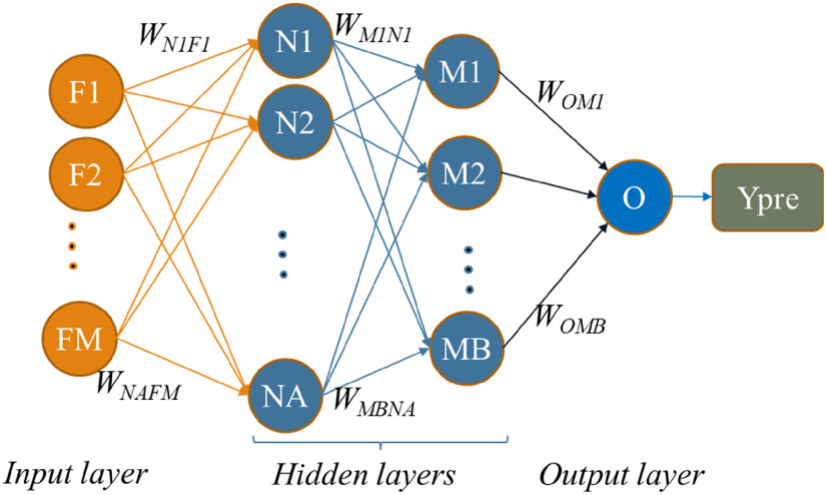
Structure of the MLP model.

In this work, we propose a federated deep learning model for STI/HIV-related tabular data. Among the different types of artificial neural network models, the multi-layered perceptron neural networks (MLPs) are exceptionally well suited for processing discrete tabular data.^26^ MLPs offer a robust solution for handling such data, given their flexibility, capacity to capture feature interactions, and widespread adoption in practical applications. They can effectively model and learn from tabular datasets, making them a valuable tool for a wide range of data analysis and machine learning tasks. A typical MLP model is described in Figure 1.

**Figure 2.**
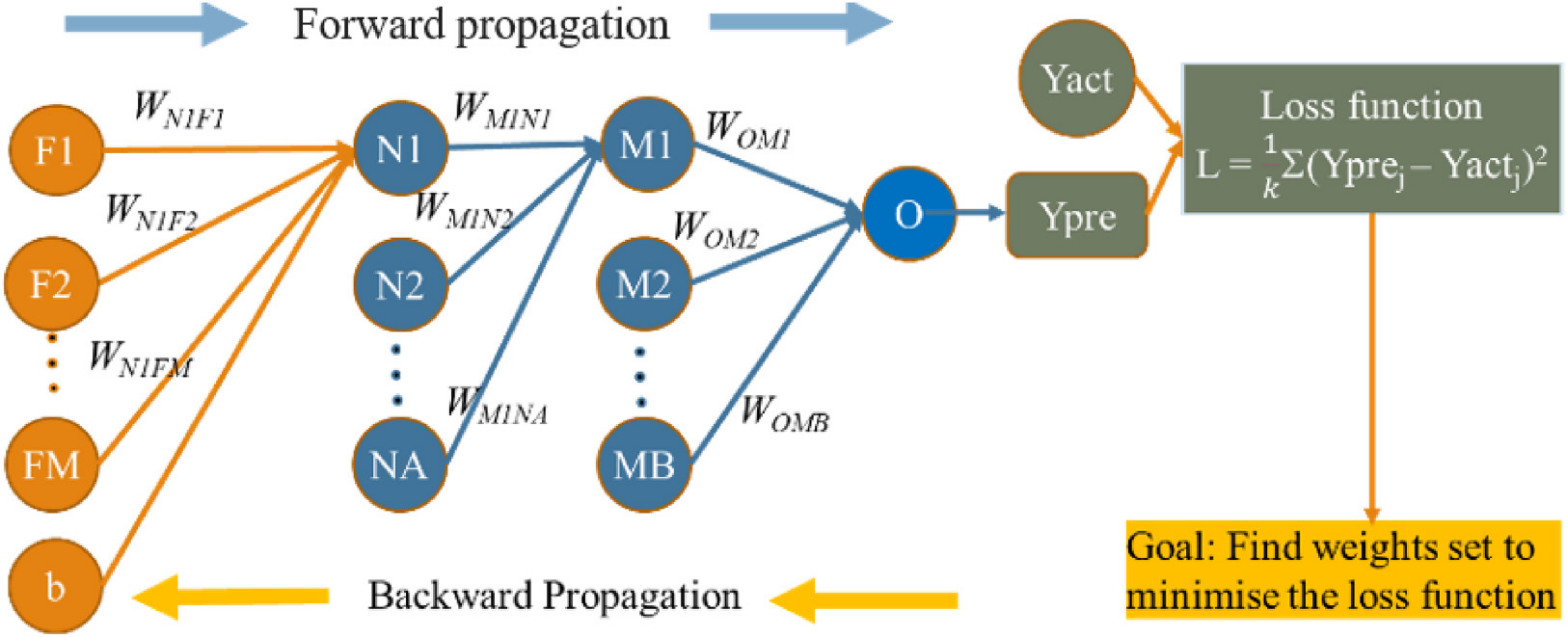
Training process of the MLP model.

A neural network model typically consists of an input layer, one or multiple hidden layers, and an output layer. Layers are interconnected with each other to all the neurons in the adjacent layers by weights. For instance, as illustrated in Figure 2, neuron N1 is linked to all input features F1 through FM.

Additionally, N1 is also connected to the subsequent hidden layer, which itself consists of MB neurons. Deep learning aims to identify the optimal set of weights for the model, thereby minimizing the error between the predicted and actual values.

The training process in MLP networks is depicted in Figure 2. Assuming that *D* is a dataset with *k* samples, we define $D=\{(x_{\dot{i}},\;y_{act_{i}}),\;\;i=1,\;2,\;\ldots ,\;k\}$ , where $x_{i}$ is the input feature vector, $x_{i}=\{F1i,\;F2i,\;\ldots FMi\}$ . The output of each neuron is calculated based on the connected inputs and corresponding weights. For instance, the output of neuron N1 is determined as:

(1) $${\mathrm{Out}}_{N1}=f\left(\sum_{i=1}^{M}W_{N1Fi}*Fi+b\right),$$

where *f* is the activation function, $W_{N1Fi}$ is the weight that presents for connection between neuron N1 and input Fi , and *b* is the bias value which may be added to each layer. The output value of neuron Ni is considered as the input value for the next layer (M). A similar process is repeated for every neuron up to the output of neuron O to get the Ypre . Based on the prediction label Ypre and the actual label Yact, the lost function is calculated. The subsequent step involves computing the derivative of the lost function and adjusting the model weights during the propagation process. The stochastic gradient descent (SGD) algorithm is consistently applied to determine the optimal weight sets for the model.^31^ In the training process within each client, a set of weights is generated. In the FL system, each client transmits its weight set to the server/cloud for aggregation, thereby forming a global model. If *Q* clients contribute to building a global model, the weights of the global model ( $W_{global}$ ) can be synthesized using the average algorithm as outlined below:

(2) $$W_{global}=\sum_{i=0}^{Q}\beta_{i}*W_{client_{i}}$$

where $\beta_{i}$ and $Wclient_{i}$ are the aggregated coefficient and set of weight of client *i*, respectively.

Once the global model is learnt, it is distributed to all clients within the FL system. The entire process of FL ensures that the original dataset of each client is not shared. While this technique safeguards data privacy to a certain extent, it is important to acknowledge that model updates exchanged during the training process may contain sensitive information, introducing the risk of privacy breaches. The challenge lies in ensuring that no identifiable information is disclosed during these updates, necessitating careful design and presenting potential challenges for the FL system. HE emerges as a suitable tool to address these limitations. By establishing a privacy-preserving environment for model training and ensuring secure model aggregation, HE enhances the robustness and privacy compliance of FL, particularly in the context of sensitive health data.

### Homomorphic encryption

Homomorphic encryption refers to encrypting data that is already encrypted rather than the original data while delivering the outcome in the same manner as it would with plaintext. This method allows intricate mathematical operations to be executed on the ciphertext without altering the encryption's fundamental characteristics.^32,33^ HE establishes a secure setting where operations can be conducted on previously encrypted data, yielding identical outcomes to those generated from the original data. Several homomorphic algorithms utilize asymmetric key systems, including RSA, ElGamal, and Paillier algorithms, along with diverse HE schemes such as Brakerski-Gentry-Vaikuntanathan (BGV), enhanced homomorphic cryptosystem (EHC), algebra homomorphic encryption scheme based on updated ElGamal (AHEE), and non-interactive exponential homomorphic encryption scheme (NEHE).^33^

Among various HE encryption algorithms, Paillier encryption is one of the optimal solutions to protect the STI/HIV risk prediction model since it is built on the security of the decisional composite residuality assumption (DCRA), which is considered a strong foundation for encryption.^34^

**Figure 3.**
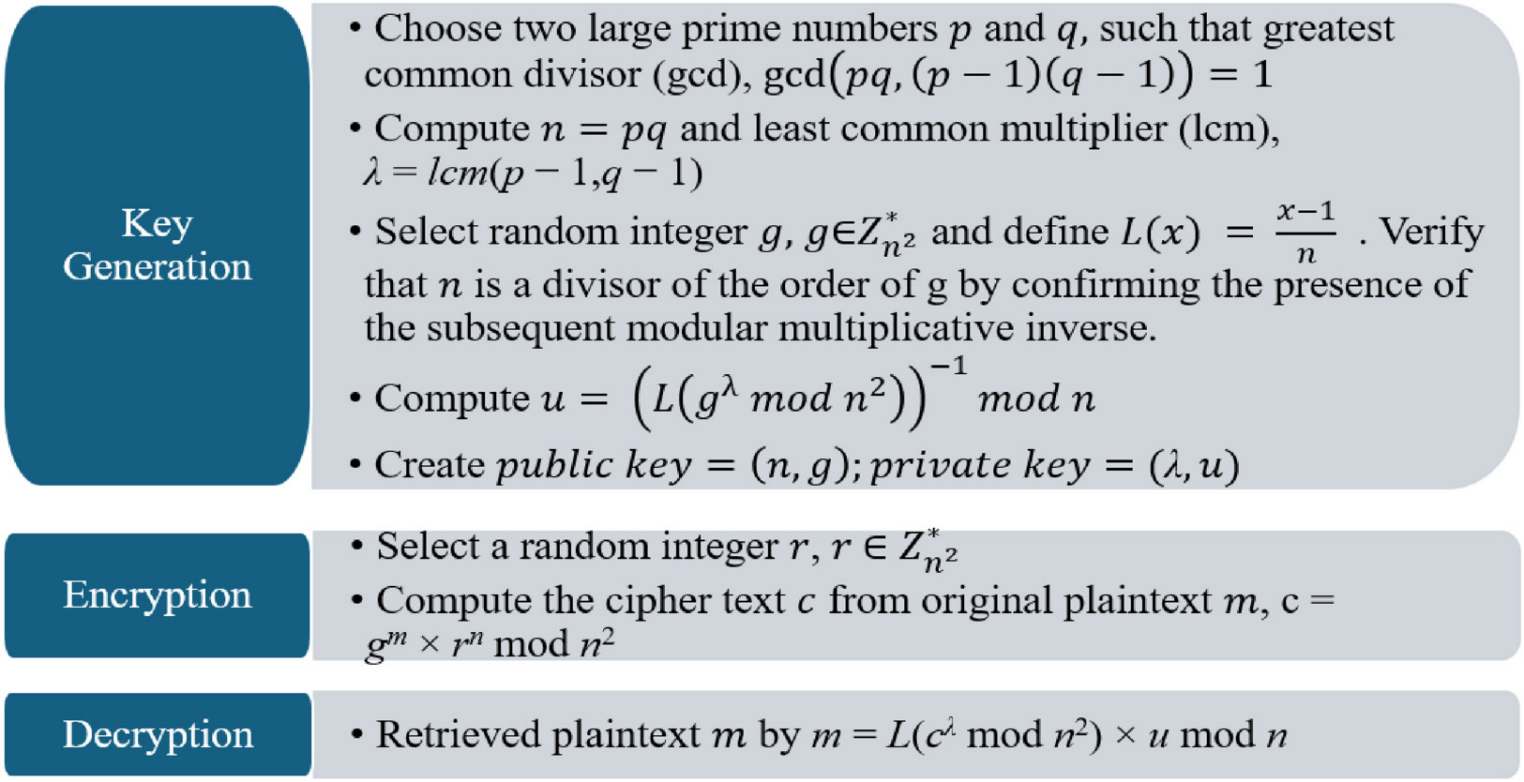
Paillier cryptosystem steps.

The Paillier encryption algorithm consists of three main steps: key generation, encryption, and decryption. The algorithm for each step is described in Figure 3.

The key generation created the public key and private key. The public key is used for encryption. Specifically, it includes the modulus n and a public generator ∈Z_(n^2)^*. These parameters are used in the encryption process to conceal the plaintext. The private key is used to retrieve the plaintext.^35^

When using the Paillier HE for a FL system, the public key and private key must be shared among clients, while the server or cloud remains unaware of the private key. Consequently, all computations on the server are performed on encrypted models or ciphertext received from the clients. Further details regarding the proposed dropout-tolerant model FLHE (DTM-FLHE) and performance enhanced FLHE (PE-FLHE) for STI/HIV risk prediction are outlined in the next section.

## Methods

### Proposed system framework

**Figure 4.**
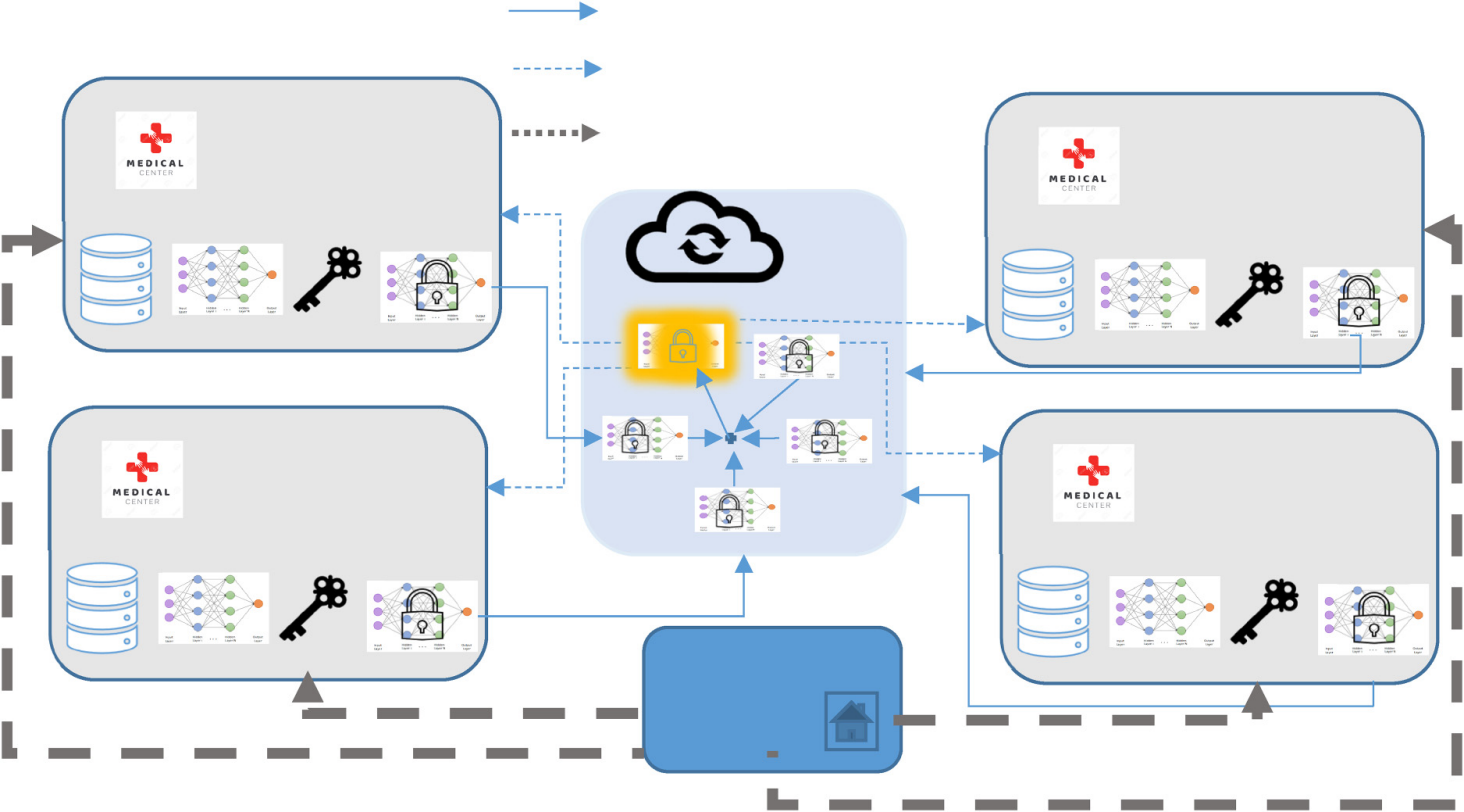
Proposed system with FLHE for STI/HIV data.

We employed FL in combination with HE for STI/HIV prediction to train deep learning models on decentralized data while upholding rigorous privacy. The FLHE system for STI/HIV risk prediction is described in this section. The system involves three key participants: clients (local data center), cloud (model aggregation site), and the key generation center. The detailed architecture of the proposed system is illustrated in Figure 4.

The key generation center generates public and private keys and distributes them to all clients. An independent key generation center within the FLHE system presents many invaluable benefits, each contributing significantly to the overall robustness and security of the data transmission and collaborative processes. Paillier encryption provides an additional layer of security, making it difficult for unauthorized parties to access or manipulate sensitive information during the FL process. Moreover, Paillier encryption is partially homomorphic, allowing for the addition of encrypted values and multiplication of an encrypted value by a plaintext constant.^36^ This property is beneficial for aggregating encrypted model updates from multiple participants in FL without the need to decrypt the individual contributions. By maintaining a distinct entity solely responsible for key generation, the system instills confidence among participating clients and the model aggregation site, assuring them of the integrity and credibility of the encryption mechanism. Moreover, this centralized approach facilitates efficient key distribution and ensures uniformity in the key generation process, thereby simplifying the administration and maintenance of the encryption framework.

The responsibility for model quality measurement typically lies with a central coordinating server, that is, the key generation center (KGC). The KGC is responsible for setting and distributing the threshold value for model quality to each client. This threshold is determined based on the specific requirements from an application and empirical analysis of model performance metrics such as AUC.

The threshold value is carefully chosen through historical data analysis and expert domain knowledge. It must be high enough to ensure the quality and robustness of the aggregated model yet balanced to allow for sufficient client participation and model diversity.

If a client's model quality falls below the specified threshold in a given round, the client is temporarily excluded from the aggregation process for that round. However, this exclusion is not permanent. The client can participate in subsequent rounds once their model quality improves and meets the required threshold. This dynamic participation mechanism ensures all clients can contribute to the FL process while maintaining the overall model quality and performance. The KGC monitors the performance continuously and adjusts thresholds as needed to optimize both the security and effectiveness of the FL system.

The model aggregation site collects encrypted models from clients and performs different secured aggregations to create the global model. The encrypted global model is then sent to clients for usage purposes. The model aggregation site does not know keys – it only does the aggregation process. It is imperative to emphasize that the model aggregation site remains devoid of direct access to the encryption keys, functioning solely to orchestrate the aggregation process. This deliberate separation of roles fortifies the security infrastructure, shielding sensitive information from unauthorized access and ensuring the integrity of the system's operations. During the exchange of information between server and clients, the system needs to fulfil some security constraints to protect the privacy of the system as follows:

1. The server is limited to obtaining encrypted models and associated secure parameters provided by the clients without access to the original local model and medical data. Nevertheless, it retains the capability to generate an aggregated global model.
2. During the local training process, clients are restricted from accessing authentic models of other clients, except for their local models and the aggregated global model. This ensures the protection of sensitive raw data belonging to other clients.

### Proposed solution to enhance accuracy

The model is named performance FLHE (PE-FLHE). To begin with, the model structure and type for the FL system are confirmed among the clients. The key generation center produces a pair of keys, namely, the publickey(n,g) and the privatekey(λ,u) , where the values of *n*, *g*, *λ*, and *u* are defined in Figure 3. These keys are distributed to all clients within the FL system. After the client model is created within each client's local data center, its weights are encrypted using the public key described below:

(3) $$C_{client_{i}}=\;g^{Wclient_{i}}\;\times r^{n}mod\;n^{2},$$

where *r* is a random integer and $r\;\in Z_{n^{2}}*$ , $C_{client_{i}}$ is the encrypted weights set of client *i* and $Wclient_{i}$ is the original weights set of client *i*. $C_{client_{i}}$ is then sent to the server/cloud for aggregation process, assuming that there are *Q* clients participating into aggregation, to create a global model. The encrypted global model is calculated as follows:

(4) $$C_{global}=\;\frac{1}{Q}\sum_{i=1}^{Q}C_{client_{i}}\;.$$

At the client side, the encrypted global model is re-aggregated with the encrypted local model before being utilized by the local client. The final FL model $\left(W_{FL_{i}}\right)$ is determined as follows:

(5) $$W_{FL_{i}}=L\left({\left(\frac{C_{global}+C_{client_{i}}}{2}\right)}^{\lambda }mod\;n^{2}\right)\;\times u\;mod\;n.$$

Our method of aggregating the local model allows it to adapt more effectively to local situations, enabling the final FL model to attain higher accuracy.

### Proposed solution to reduce computational cost

To implement a federated learning with homomorphic encryption (FLHE), each client is required to encrypt its local model and decrypt the global model, which can result in reducing significant computational overhead for the entire system. Addressing this issue, a client dropout technique has emerged as a promising solution to enhance the computational efficiency of the system. An illustrative approach, such as the dropout-tolerant participants based on the Diffie-Hellman and Shamir secret sharing algorithm presented in reference,^16^ is a viable method to mitigate the computation cost.

Dropout-tolerant model FLHE (DTM-FLHE) introduces a client-dropout solution that ensures only high-quality models contributing to the FL process. Specifically, if the quality of a client model, measured by metrics such as the AUC, falls below a predetermined threshold, the client is temporarily excluded. The DTM-FLHE framework employs HE to maintain data privacy during training, allowing secure aggregation of model parameters without revealing sensitive information. This adaptive approach dynamically adjusts client participation based on real-time performance metrics, enhancing the overall accuracy and robustness of the global model. Avoiding incorrect threshold settings could lead to the exclusion of potentially valuable data from clients that might perform better in subsequent rounds. In that case, the client is spared from encrypting and transmitting its model to develop the global model in the round, which does not have good AUC. This adaptive approach not only alleviates the computational load on underperforming clients but also optimizes the allocation of computational resources, ultimately enhancing the overall accuracy and dependability of the final global model.

## Results

### Dataset

The data were sourced from the Demographic and Health Surveys (DHS). Our study utilized 168,459 data entries from eight distinct countries collected between 2013 and 2018, as presented in Table 1.

**Table 1.** Dataset characteristics.

Country	The number of Samples Collected	Data Collection Year
Dominica	9717	2013
Dominican Republic	2028	2013
India	107,297	2015
Haiti	9572	2016
Guinea	3831	2018
Guinea	3688	2012
Ethiopia	11,327	2016
Cameroon	6648	2018
Angola	50,150	2015

The model was trained using the FL framework, with each client trained locally on its dataset for a specified number of epochs before averaging the model weights. The training procedure involved 50 communication rounds, with each client performing five local epochs per round. The learning rate was set to 0.01, and the batch size was 32. The DHS program encompassed information on behaviors, clinical tests for STI/HIV, and demographic data related to men's health.

The selection of features was based on a comprehensive analysis of relevant literature^37–39^ and consultations with professionals in public and sexual health. The input features included *age, education level, wealth index, regionality, condom use during last sex with a recent partner, current marital status, age at first sexual intercourse, recent sexual activity, always use condoms during sex, has one sexual partner, number of lifetime sexual partner, given gifts or other goods in exchange for sex*, and *if a woman was justified in asking for condom use if the sexual partner/s has an STI*.

Due to the highly imbalanced nature of the dataset, the oversampling technique was implemented to address this disparity. By synthetically increasing the instances of the minority class, the dataset was rebalanced, ensuring that the model was not biased toward the majority class during the training process. This approach facilitated a more comprehensive learning process, enabling the model to capture the intricacies of both classes and make more accurate predictions.

Once the dataset was rebalanced through oversampling, the training commenced on this more equitable data representation. This allowed the model to learn from more diverse examples, enhancing its ability to discern subtle patterns and make informed predictions on both classes. By training on the balanced dataset, the model was better equipped to generalize its learning and make more reliable predictions when presented with new, unseen data points.

### Model selection

In this study, we used the MLP model because this type of model has proven to be a resilient option for managing this type of data because of its adaptability, capability to comprehend feature relationships, and extensive integration in real-world scenarios. Their adeptness in representing and comprehending tabular datasets makes them a valuable asset for diverse data analysis and machine learning responsibilities.^31^

 After optimizing to identify the most suitable model for this task, we settled on an MLP model that incorporated 13 input features, two hidden layers (with eight and four neurons, respectively), and the output layer. Following establishing the model structure, we determined the optimal activation function and optimizer for this specific dataset. The selected activation function was the sigmoid function $\left(f(x)=1\;/\;(1+e^{-x})\right)$ . This function is widely renowned for its effectiveness in predicting the probability of the output,^40^ particularly in the context of STI/HIV risk, where the probability typically ranges between 0 and 1. This specific function consistently demonstrated favorable outcomes after conducting several experiments and comparing the results with alternative activation functions. Similarly, we applied a comparable process with various optimizers, ultimately selecting the Adam optimizer as the most optimal choice.

### Training procedure and performance metrics

Our model was trained using the proposed framework. Each client model was trained locally on its dataset for a specified number of epochs before averaging the model weights. The training procedure in this study involved 40 communication rounds, each client performing five local epochs per round. The learning rate was set to 0.01, and the batch size was 64. The model was compiled with the binary cross-entropy loss function and the NAdam optimizer. The AUC and accuracy were considered to be the evaluation metrics.

## Results

In this section, we compare our proposed solutions for applying FLHE in STI/HIV risk prediction with the original FLHE, FL, and centralized data models.

### Model performance evaluation of different solutions for predicting HIV risk

We compared our proposed solutions in terms of AUC and accuracy with different strategies and model structures, including local training,^41^ centralized learning,^42^ and FLHE,^27^ for predicting HIV risk, as outlined in Table 2. The data for each country was split into two groups, with 70% being allocated for training and 30% for testing. The local model signifies a model trained solely on local data. FL represents the model aggregated from all local models. Centralized learning refers to the model constructed from the centralized data, where each client contributes 70% of the data to train the centralized model. FLHE denotes the model created through a combination of the HE technique and FL. DTM-FLHE represents the FLHE-dropout model, which is from the result of combining our proposed dropout technique with FLHE and designed to enhance computational efficiency. Finally, PE-FLHE depicts the performance enhancement FLHE model, which is our solution to improving accuracy by conducting a secondary aggregation with the local model before used with local data. The average AUC and accuracy results for each type of model are also presented in Table 2

**Table 2.** Performance of different solutions for predicting HIV risk.

Countries	Local Model		FL		Centralized Learning		FLHE		DTM-FLHE		PE-FLHE	
	AUC	Accuracy	AUC	Accuracy	AUC	Accuracy	AUC	Accuracy	AUC	Accuracy	AUC	Accuracy
Dominican	0.77	78.2	0.97	93.0	0.99	92.0	0.96	92.1	0.96	93.0	0.96	93.0
Dominican Republic	0.81	75.0	0.97	87.5	0.97	90.0	0.95	92.6	0.95	92.0	0.95	92.0
India	0.61	60.0	0.91	86.2	0.92	83.0	0.93	87.0	0.92	86.6	0.93	88.0
Haiti	0.72	64.3	0.87	77.6	0.81	76.0	0.83	78.2	0.8	76.1	0.88	82.0
Guinea	0.84	71.4	0.96	92.9	0.92	92.0	0.94	93.5	0.94	92.2	0.98	96.2
Ethiopia	0.94	92.2	0.96	94.7	0.96	91.0	0.95	91.1	0.95	92.0	0.97	94.6
Cameroon	0.68	70.2	0.81	72.3	0.81	77.0	0.8	80.5	0.8	79.0	0.89	87.1
Angola	0.88	84.4	0.9	80.0	0.86	86.0	0.9	88.1	0.89	86.8	0.94	92.7
Average	0.78	74.5	0.92	85.5	0.91	85.9	0.91	87.9	0.90	87.2	0.94	90.7

The first point is that the local model demonstrates relatively weaker performance, with an AUC of 0.78 and an accuracy of 74.4%. This suggests that the model may not effectively distinguish between individuals at higher and lower risk of HIV, indicating limitations in capturing the complexities of the data and making accurate predictions.

The FL model showed a significant improvement over the local model, with an AUC of 0.92 and an accuracy of 85.5%. This suggests that the FL model is more effective in differentiating between individuals at varying risk levels for HIV. The higher accuracy rate implies that the FL model can make precise predictions for a substantial portion of the dataset, demonstrating its reliability for HIV risk assessment.

The centralized learning model produced similar results to FL, with an AUC of 0.9 and an accuracy of 85.8%. The centralized learning model shows solid performance, although it slightly lags behind the FL model. This indicates that a centralized approach can be effective. However, it may not be as efficient as the FL approach, particularly when dealing with sensitive data or in scenarios where data privacy is crucial.

The FLHE model achieved an AUC of 0.9 and an accuracy of 87.8%. This performance showcases the successful integration of privacy-preserving techniques such as HE within the FL framework. It allows for collaborative model training on the encrypted data, ensuring data privacy while achieving high predictive accuracy.

**Figure 5.**
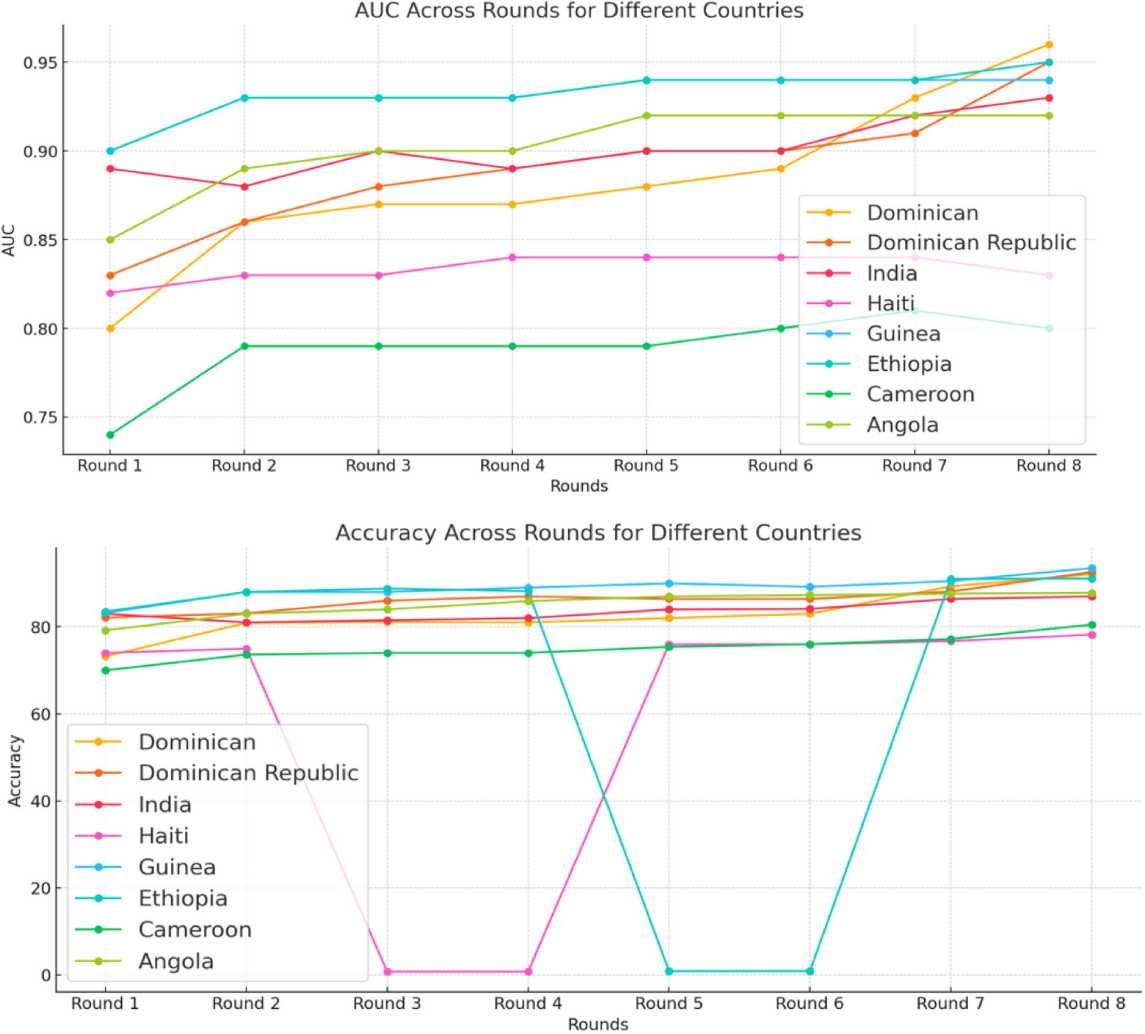
Performance of FLHE across eight rounds.

The FLHE performance for different rounds is illustrated in Figure 5. The first plot shows the trends in AUC values for each country across the eight rounds. The AUC results show consistently improved AUC over the rounds, with some fluctuations, for the most countries. The second plot shows the trends in accuracy values for each country across the eight rounds. There is a general upward trend for the results of accuracy. These plots illustrate how the FLHE model's performance evolves over time, highlighting improvements.

The proposed *DTM-FLHE* exhibits competitive performance, boasting an AUC of 0.9 and an accuracy of 87.2%. By integrating dropout techniques for lower-quality client models within the FLHE framework, this model enhances its robustness and predictive capabilities, simultaneously reducing the overall computational cost. Although the AUC remains consistent with the FL and centralized learning models, a slight decrease in accuracy suggests a potential trade-off between the complexity of the model and its predictive accuracy.

The proposed *PE-FLHE* with accuracy enhancement stands out as the top performer, with an AUC of 0.94 and an accuracy of 90.7%. This significant improvement in AUC and accuracy demonstrates the incorporation of advanced techniques, such as feature engineering and model fine-tuning, leading to superior predictive performance. The high accuracy rate indicates the model's strong capability in identifying individuals at different risk levels for HIV, making it a robust tool for effective risk assessment and intervention strategies.

**Figure 6.**
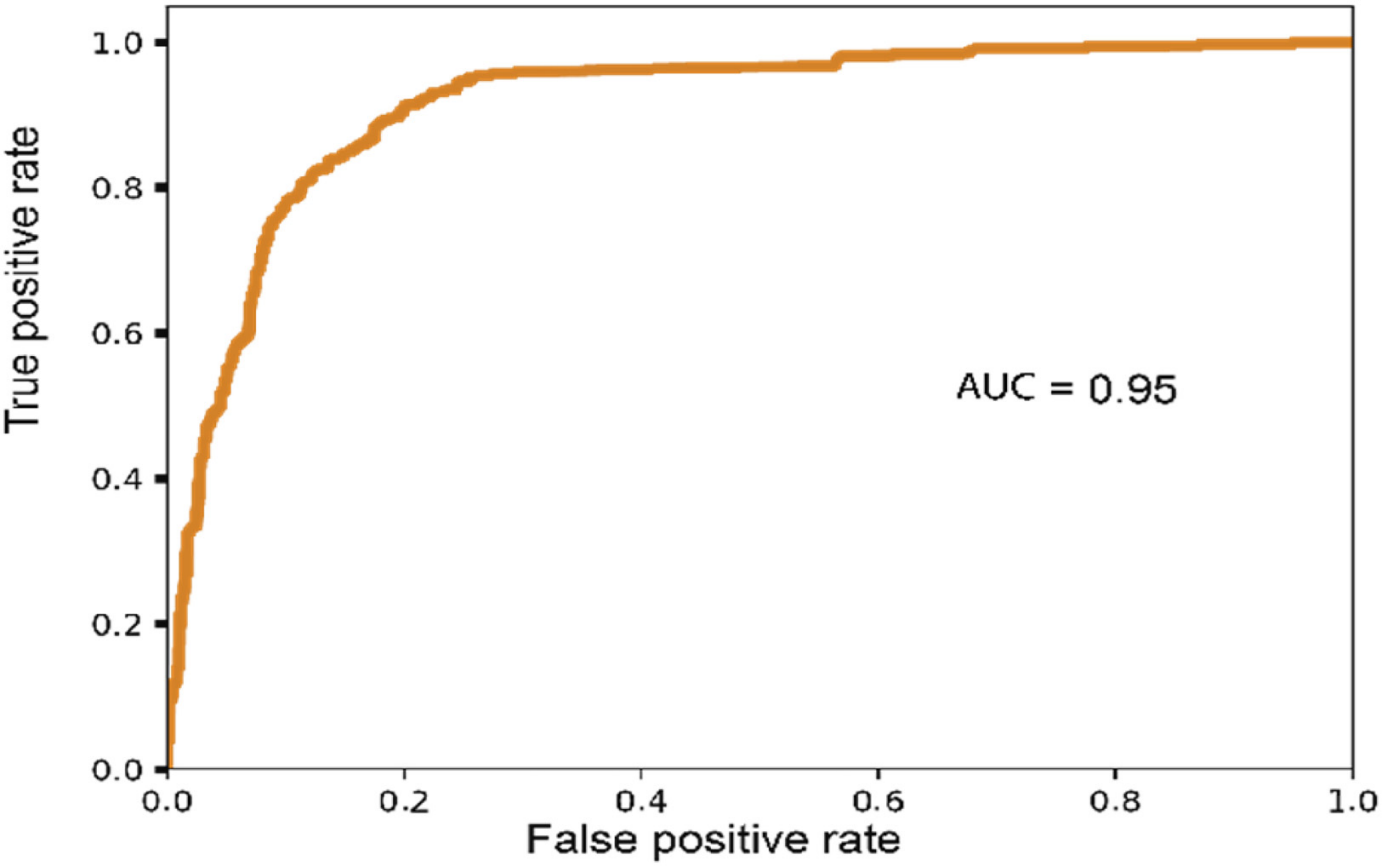
AUC of Dominican Republic under the PE-FLHE method.

We have closely examined the Dominican Republic case and present the AUC results for HIV risk prediction in the Dominican Republic which are depicted in Figure 6.

The comparison highlights the substantial benefits of the FL and HE solutions in enhancing the predictive performance of models, particularly in cases where data privacy and security are paramount. The results emphasize the importance of collaborative approaches such as FL and advanced privacy-preserving techniques such as HE in effectively analyzing sensitive data, ensuring accurate predictions, and enabling valuable insights in the context of the Dominican Republic data.

The average result across different countries emphasizes the potential for incorporating sophisticated techniques and model enhancements, as demonstrated by the proposed FLHE model, with an accuracy enhancement and dropout model, to improve the model's predictive capabilities and overall performance significantly.

### Model performance evaluation for predicting STI risk

We conducted similar experiments using data from eight countries to predict the risks of sexually transmitted infections (STIs), as presented in Table 3. While the overall performance of the prediction models for STI risk is somewhat lower compared to the prediction for HIV risk, we still observed consistent trends. In other words, FL and our proposed models (*DTM-FLHE* and *PE-FLHE*) demonstrate notable security and computational efficiency enhancements.

**Table 3.** Performance of different solutions for predicting STIs risk.

Countries	Local Learning		FL		Centralized Learning		FLHE		DTM-FLHE		PE-FLHE	
	AUC	Accuracy	AUC	Accuracy	AUC	Accuracy	AUC	Accuracy	AUC	Accuracy	AUC	Accuracy
Dominican	0.79	70.1	0.82	73.2	0.79	73.0	0.84	76.1	0.83	76.1	0.81	74.2
Dominican Republic	0.83	76.5	0.86	79.6	0.86	80.0	0.87	83.5	0.87	83.0	0.89	83.2
India	0.62	60.0	0.63	60.0	0.63	65.0	0.64	60.0	0.62	60.0	0.63	60.0
Haiti	0.68	62.3	0.69	63.0	0.68	65.0	0.70	65.1	0.70	65.1	0.71	66.2
Guinea	0.78	71.0	0.79	75.1	0.80	76.7	0.80	74.5	0.80	75.1	0.80	74.0
Ethiopia	0.82	74.1	0.83	76.1	0.82	77.5	0.84	77.3	0.81	75.8	0.85	78.3
Cameroon	0.74	67.5	0.74	70.0	0.74	70.2	0.74	69.1	0.74	70.0	0.76	70.0
Angola	0.79	68.9	0.79	71.3	0.83	70.0	0.78	73.0	0.77	72.0	0.78	73.6
Average	0.76	68.8	0.77	71.0	0.77	72.2	0.78	72.3	0.77	72.1	0.78	72.4

As demonstrated in Table 3, the proposed methods (DTM-FLHE and PE-FLHE) generally outperform local learning, traditional FL, and centralized learning in terms of both AUC and accuracy. For instance, in the Dominican Republic case, the DTM-FLHE model achieves an AUC of 0.87 and an accuracy of 83.00%, while the PE-FLHE model reaches an AUC of 0.89 and an accuracy of 83.20%. These improvements highlight the effectiveness of our approaches in leveraging FL with enhanced privacy-preserving mechanisms, resulting in better predictive performance and greater security.

## Discussion

This research introduces a novel solution called the *DTM-FLHE* for enhancing the computational efficiency of the system implementing FLHE.

Our method employs client-dropout based on model quality to manage the computational overhead caused by the encryption and transmission of models in FLHE.

If the quality of a client model, measured by metrics such as the AUC, falls below a specified threshold, the client is excluded from contributing its model to the global model development. This adaptive strategy not only reduces the computational load on underperforming clients but also optimizes the allocation of computational resources, ultimately leading to an improved global model's accuracy and reliability.

Comparative analysis was conducted to assess the efficacy of the proposed *DTM-FLHE* model in STI/HIV risk prediction alongside the original FLHE, FL, and centralized data models. The results reveal an AUC of 0.9 and an accuracy of 87.2% for the *DTM-FLHE* model, demonstrating its efficiency in balancing computational costs without significantly compromising predictive accuracy.

The method proposed for *PE-FLHE* involves the secure encryption of the model structural weights using a public key and the subsequent aggregation of the encrypted client models to form a global model, which is then shared among clients. This approach allows for the efficient adaptation of the local model to its specific context, enhancing the overall accuracy of the final FL model. The results indicate a significant improvement in model performance, with an impressive AUC of 0.94 and an accuracy of 90.7%. These results highlight the efficacy of the proposed approach in achieving higher predictive accuracy, especially in the challenging task of STI/HIV risk predictions.

Compared to other methods, the *PE-FLHE* method demonstrates a superior AUC and accuracy compared to the original FLHE, FL, and centralized data models. Specifically, it outperforms the FL model with an AUC of 0.91 and the centralized learning model with an AUC of 0.92, underscoring its effectiveness in handling the complexities of the data and improving predictive capabilities. Additionally, the enhanced accuracy of the *PE-FLHE* method surpasses the AUC of 0.9 achieved by the FLHE-dropout model, reinforcing its superiority in ensuring precise predictions and robust performance.

The proposed method's capability to adapt to the local data nuances sets it apart from other approaches, enabling it to capture the intricacies of the Dominican dataset more effectively. By optimizing the aggregation process and leveraging the encrypted global model, the *PE-FLHE* method effectively balances computational costs while enhancing the final model's accuracy. These findings emphasize the significance of incorporating adaptive strategies within the FLHE framework to achieve higher predictive accuracy, thus demonstrating the method's potential for improving healthcare data analysis and risk prediction in complex scenarios such as STI/HIV assessments.

### Limitations of study

Despite employing client dropout strategies to reduce computational overhead, the encryption and decryption processes inherent in HE remain computationally intensive. This could limit the system's scalability, particularly for clients with limited computational resources. The proposed method uses a specified threshold for client dropout based on model quality metrics such as AUC. However, this process of determining and dynamically adjusting these thresholds needs to be experimented with more diversified data.

## Conclusion

The study presents an innovative approach that combines FL and HE for STI/HIV prediction. This system provides a robust framework for training deep learning models on decentralized data while ensuring stringent privacy measures. Additionally, the study outlined a strategy to enhance model performance evaluated using AUC and accuracy metrics, which involved a secondary aggregation at the local level before utilizing the global model for each client. By employing FL and homomorphic encryption, the security of the entire system was effectively maintained. Furthermore, we implemented a novel dropout approach as a viable solution for reducing computational costs on the client side. This approach involved setting a threshold for model quality (e.g., AUC) for each client. If the client's model falls below the specified threshold, there is no need to encrypt and transmit it to the server for aggregation. In addition, we provide a comparative analysis of different methodologies for constructing deep learning models in the context of STI/HIV prediction. A thorough evaluation of the strengths and weaknesses of each approach serves as a valuable benchmark point for future applications and research in this research field.

Despite the variations in performance across different countries, the trend remains clear, which is that our proposed models consistently provide superior results, demonstrating their robustness and adaptability in diverse contexts. This reinforces the potential for advanced FL techniques in improving the accuracy and reliability of health risk predictions while maintaining stringent security standards.

For future research, exploring advanced techniques to enhance the privacy preservation mechanisms in FL and HE is suggested. Investigating the applications of differential privacy and other state-of-the-art cryptographic protocols could potentially enhance the existing privacy framework. Moreover, conducting extensive real-world experiments and validations on larger and more diverse datasets can help verify the scalability and robustness of the proposed methodology. Lastly, developing a comprehensive framework for incorporating various models and integrating them seamlessly within the FL context can be a promising direction for future investigations in this area.

## Supplemental Material

sj-pdf-1-dhj-10.1177_20552076241298425 - Supplemental material for High security and privacy protection model for STI/HIV risk prediction Supplemental material, sj-pdf-1-dhj-10.1177_20552076241298425 for High security and privacy protection model for STI/HIV risk prediction by Zhaohui Tang, Thi Phuoc Van Nguyen, Wencheng Yang, Xiaoyu Xia, Huaming Chen, Amy B. Mullens, Judith A. Dean, Sonya R Osborne and Yan Li in DIGITAL HEALTH

## References

[1] Besoain F Perez-Navarro A Jacques Aviñó C , et al. Prevention of HIV and other sexually transmitted infections by geofencing and contextualized messages with a gamified app, UBESAFE: design and creation study. JMIR Mhealth Uhealth 2020; 8: e14568. 10.2196/14568 PMC7109613 32181752

[2] Moore QL Paul ME McGuire AL , et al. Legal barriers to adolescent participation in research about HIV and other sexually transmitted infections. Am J Public Health 2016; 106: 40–44. 26562103 10.2105/AJPH.2015.302940 PMC4695940

[3] Yamakawa M Shiina T Nishida N , et al. Computer aided diagnosis system developed for ultrasound diagnosis of liver lesions using deep learning, in 2019. In: 2019 IEEE International Ultrasonics Symposium (IUS), Glasgow, Scotland, 6–9 October, 2019, pp. 2330–2333.

[4] Srivastava S Soman S Rai A , et al. Deep learning for health informatics: recent trends and future directions. In: 2017 international conference on advances in computing, communications and informatics (ICACCI), Manipal University, Karnataka, India, 13–16 September, 2017, pp. 1665–1670.

[5] Santangelo OE Gentile V Pizzo S , et al. Machine learning and prediction of infectious diseases: a systematic review. Mach Learn Knowl Extr 2023; 5: 175–198.

[6] Rajendran NM Karthikeyan M Raja K , et al. Communicable disease prediction using machine learning and deep learning algorithms. In: International conference on information, communication, and computing technology (ICICCT) 2023, Jagan Institute of Management Studies in Rohini, New Delhi, India, 27 May, 2023. Springer.

[7] Houssein EH Sayed A . Boosted federated learning based on improved particle swarm optimization for healthcare IoT devices. Comput Biol Med 2023; 163: 107195. 37393788 10.1016/j.compbiomed.2023.107195

[8] Nguyen DC Pham VQ Pathirana PN , et al. Federated learning for smart healthcare: a survey. ACM Comput Surv (CSUR) 2022; 55: 1–37.

[9] Xu J Glicksberg BS Su C , et al. Federated learning for healthcare informatics. J Healthc Inform Res 2021; 5: 1–19. 33204939 10.1007/s41666-020-00082-4 PMC7659898

[10] Rani S Kataria A Kumar S , et al. Federated learning for secure IoMT-applications in smart healthcare systems: a comprehensive review. Knowl Based Syst 2023; 274: 110658.

[11] Pandya S Srivastava G Jhaveri R , et al. Federated learning for smart cities: a comprehensive survey. Sustain Energy Technol Assessments 2023; 55: 102987.

[12] Chowdhury A Kassem H Padoy N , et al. A review of medical federated learning: applications in oncology and cancer research. In: 7th international MICCAI Brainlesion workshop (BrainLes 2021), Strasbourg, France, 27 September, 2021. Springer.

[13] Chen M Yang Z Saad W , et al. A joint learning and communications framework for federated learning over wireless networks. IEEE Trans Wireless Commun 2020; 20: 269–283.

[14] Hu H Salcic Z Sun L , et al. Membership inference attacks on machine learning: a survey. ACM Comput Surv (CSUR) 2022; 54: 1–37.

[15] Lyu L Yu H Ma X , et al. Privacy and robustness in federated learning: attacks and defences. IEEE Trans Neural Netw Learn Syst 2024; 35: 8726–8746. 36355741 10.1109/TNNLS.2022.3216981

[16] Zhang L Xu J Vijayakumar P , et al. Homomorphic encryption-based privacy-preserving federated learning in IoT-enabled healthcare system. IEEE Trans Netw Sci Eng 2022; 10: 2864–2880.

[17] Hardy S Henecka W Ivey-Law H , et al. Private federated learning on vertically partitioned data via entity resolution and additively homomorphic encryption. arXiv preprint arXiv:1711.10677, 2017.

[18] Ma J Naas S-A Sigg S , et al. Privacy-preserving federated learning based on multi-key homomorphic encryption. Int J Intell Syst 2022; 37: 5880–5901.

[19] Madi A Stan O Mayoue A , et al. A secure federated learning framework using homomorphic encryption and verifiable computing. In: 2021 Reconciling data analytics, automation, privacy, and security: a big data challenge (RDAAPS), Toronto, Canada, 13–15 May, 2021. IEEE.

[20] Stripelis D Saleem H Ghai T , et al. Secure neuroimaging analysis using federated learning with homomorphic encryption. In: 17th international symposium on medical information processing and analysis (SIPAIM), Campinas, Brazil, 17–19 November, 2021, pp. 351–359. SPIE.

[21] Zhang C Li S Xia J , et al. {BatchCrypt}: efficient homomorphic encryption for {Cross-Silo} federated learning. In: 2020 USENIX annual technical conference (USENIX ATC 2020), Boston, MA, USA, 15–17 July, 2020.

[22] Lu Y Fan L . An efficient and robust aggregation algorithm for learning federated CNN. In: Proceedings of the 2020 3rd international conference on signal processing and machine learning (SPML 2020), Beijing, China, 22–24 October, 2020, pp. 1–7.

[23] Silva PR Vinagre J Gama J . Towards federated learning: an overview of methods and applications. Wiley Interdiscip Rev: Data Mining Knowl Discov 2023; 13: e1486.

[24] Enthoven D Al-Ars Z . *An overview of federated deep learning privacy attacks and defensive strategies*. In: Federated learning systems: towards next-generation AI, 2021, pp. 173–196.

[25] Bhowmick A Duchi J Freudiger J , et al. Protection against reconstruction and its applications in private federated learning. arXiv preprint arXiv:1812.00984, 2018.

[26] Zhu L Liu Z Han S . Deep leakage from gradients. Adv Neural Inf Process Syst 2019; 32: 11.

[27] Fang H Qian Q . Privacy preserving machine learning with homomorphic encryption and federated learning. Future Internet 2021; 13: 94.

[28] Zia MT Khan MA El-Sayed H . Application of differential privacy approach in healthcare data—a case study. In: 2020 14th international conference on innovations in information technology (IIT), University of Sharjah, United Arab Emirates, 14–16 December, 2020. pp.35–39.

[29] Bhatt D Patel C Talsania H , et al. CNN variants for computer vision: history, architecture, application, challenges and future scope. Electronics (Basel) 2021; 10: 2470.

[30] Torres JF Hadjout D Sebaa A , et al. Deep learning for time series forecasting: a survey. Big Data 2021; 9: 3–21. 33275484 10.1089/big.2020.0159

[31] Al Bataineh A Kaur D Jalali SMJ . Multi-layer perceptron training optimization using nature inspired computing. IEEE Access 2022; 10: 36963–36977.

[32] Boneh D Goh E-J Nissim K . Evaluating 2-DNF formulas on ciphertexts. In: Proceedings of theory of cryptography: second theory of cryptography conference, TCC 2005, Cambridge, MA, USA, February 10–12, 2005. Springer, 2005.

[33] Parmar PV Padhar SB Patel SN , et al. Survey of various homomorphic encryption algorithms and schemes. Int J Comput Appl 2014; 91: 11.

[34] Paillier P . Public-key cryptosystems based on composite degree residuosity classes. In: International conference on the theory and applications of cryptographic techniques. Springer, 1999.

[35] Armknecht F Boyd C Carr C , et al., A guide to fully homomorphic encryption. Cryptology ePrint Archive, 2015.

[36] Morris L . Analysis of partially and fully homomorphic encryption. Rochester Inst Technol 2013; 10: 1–5.

[37] Bao Y Medland NA Fairley CK , et al. Predicting the diagnosis of HIV and sexually transmitted infections among men who have sex with men using machine learning approaches. J Infect 2021; 82: 48–59. 33189772 10.1016/j.jinf.2020.11.007

[38] Xu X Fairley CK Chow EPF , et al. Using machine learning approaches to predict timely clinic attendance and the uptake of HIV/STI testing post clinic reminder messages. Sci Rep 2022; 12: 8757. 35610227 10.1038/s41598-022-12033-7 PMC9128330

[39] He J Li J Jiang S , et al. Application of machine learning algorithms in predicting HIV infection among men who have sex with men: model development and validation. Front Public Health 2022; 10: 967681. 36091522 10.3389/fpubh.2022.967681 PMC9452878

[40] Sharma S Sharma S Athaiya A . Activation functions in neural networks. Towards Data Sci 2017; 6: 310–316.

[41] Fengshi J Ye Y Zhou Y , et al., Identification of key influencers for secondary distribution of HIV self-testing among Chinese MSM: a machine learning approach. Medrxiv, 2021.

[42] Mutai CK McSharry PE Ngaruye I , et al. Use of machine learning techniques to identify HIV predictors for screening in sub-Saharan Africa. BMC Med Res Methodol 2021; 21: 1–11. 34332540 10.1186/s12874-021-01346-2 PMC8325403

