# Supplementary material for: High security and privacy protection model for STI/HIV risk prediction
Source: Digit Health. 2024 Nov 21;10:20552076241298425. doi: 10.1177/20552076241298425 (PMC11580078; doi:10.1177/20552076241298425)
Supplement: sj-pdf-1-dhj-10.1177_20552076241298425 - Supplemental material for High security and privacy protection model for STI/HIV risk prediction [file sj-pdf-1-dhj-10.1177_20552076241298425.pdf]

SEARCH | LOGIN | Select Language

Search The DHS Program | Search

COUNTRIES
- Countries
- Countries List
- Interactive Map

DATA
- Data
- Using Datasets for Analysis
- Guide to DHS Statistics
- Download Datasets
- IPUMS-DHS
- DHS User Forum
- STATcompiler
- Mobile App
- DHS API
- Visualizations

PUBLICATIONS
- Publications
- Publications Search
- Journal Articles Search
- Publications Catalog

METHODOLOGY
- Methodology
- Survey Types
- Survey Characteristics Search
- Questionnaires and Manuals
- Tabulation Plan for DHS Final Report
- Tabulation Plan for Key Indicators Report
- Survey Process
- Survey Questions Database
- GIS
- Dissemination
- Capacity Strengthening

RESEARCH
- Research
- Featured Studies
- DHS Fellows Program

TOPICS
- Topics
- Childhood Mortality
- COVID-19
- Family Planning
- Gender
- Malaria
- Maternal Mortality
- Nutrition
- SDGs
- Wealth Index
- More topics

The DHS Program > Data > Datasets Account Home

Datasets

- **My Account**
- Approved Countries
- Update Personal Information
- Change Password
- Change Email
- Logout

My Dataset Account

Logged in: phuocvan.haui@gmail.com

Approved Countries by Project and Dataset Type

AI assistant to predict HIV STI (submitted: 04/06/2022)

Approved countries:

Angola	SURVEY, HIV
Armenia	SURVEY
Bolivia	SURVEY
Brazil	SURVEY
Burkina Faso	SURVEY, HIV
Burundi	SURVEY, HIV
Cambodia	SURVEY, HIV
Cameroon	SURVEY, HIV
Chad	SURVEY, HIV
Colombia	SURVEY
Comoros	SURVEY
Congo	SURVEY, HIV
Congo Democratic Republic	SURVEY, HIV
Cote d'Ivoire	SURVEY, HIV
Dominican Republic	SURVEY, HIV
Ecuador	SURVEY

Egypt	SURVEY, HIV
El Salvador	SURVEY
Eswatini	SURVEY, HIV
Ethiopia	SURVEY, HIV
Gabon	SURVEY, HIV
Gambia	SURVEY, HIV
Ghana	SURVEY, HIV
Guatemala	SURVEY
Guinea	SURVEY, HIV
Haiti	SURVEY, HIV
India	SURVEY, HIV
Jordan	SURVEY
Kazakhstan	SURVEY
Kenya	SURVEY, HIV
Kyrgyz Republic	SURVEY
Lesotho	SURVEY, HIV
Liberia	SURVEY, HIV
Malawi	SURVEY, HIV
Mali	SURVEY, HIV
Mexico	SURVEY
Mozambique	SURVEY, HIV
Namibia	SURVEY, HIV
Niger	SURVEY, HIV
Nigeria	SURVEY
Nigeria (Ondo State)	SURVEY
Papua New Guinea	SURVEY
Peru	SURVEY
Rwanda	SURVEY, HIV
Sao Tome and Principe	SURVEY, HIV
Senegal	SURVEY, HIV
Sierra Leone	SURVEY, HIV
South Africa	SURVEY, HIV
Tajikistan	SURVEY
Tanzania	SURVEY, HIV
Togo	SURVEY, HIV
Tunisia	SURVEY
Turkey	SURVEY
Uganda	SURVEY, HIV
Ukraine	SURVEY
Uzbekistan	SURVEY
Vietnam	SURVEY, HIV
Zimbabwe	SURVEY, HIV

Interactive Map

Capacity Strengthening

RESEARCH

Contraceptive Profiles
DHS Fellows Program

Mobile App
DHS API
Visualizations

PUBLICATIONS

Publications Search
Journal Articles Search
Publications Catalog

SDGs
More Topics

ICF, 530 Gaither Road, Suite 500, Rockville, MD 20850
Tel: +1 301 407-6500 * Fax: +1 301 407-6501

The information provided on this Web site is not official U.S. Government information and does not represent the views or positions of the U.S. Agency for International Development or the U.S. Government.
